# Ethical Leadership and Followers’ Emotional Exhaustion: Exploring the Roles of Three Types of Emotional Labor toward Leaders in South Korea

**DOI:** 10.3390/ijerph182010862

**Published:** 2021-10-15

**Authors:** Hyewon Lee, Saemi An, Ga Young Lim, Young Woo Sohn

**Affiliations:** Psychology Department, Yonsei University, Seoul 06695, Korea; julyhyewon@yonsei.ac.kr (H.L.); saemi08@yonsei.ac.kr (S.A.); gylim@yonsei.ac.kr (G.Y.L.)

**Keywords:** emotional exhaustion, ethical leadership, emotional labor, intra-organizational emotion regulation, conservation of resources theory

## Abstract

Employees’ emotional exhaustion caused by their leaders has significant consequences for both individuals and organizations. Identifying the roles of intra-organizational emotional labor is important to prevent employees’ emotional exhaustion. This study examined the relationships between ethical leadership, followers’ emotional labor toward leaders, and emotional exhaustion using Hobfoll’s conservation of resources theory. Data collected from 259 employees working in South Korea were analyzed using regression and SEM. The results indicate that ethical leadership was negatively related to followers’ emotional exhaustion. It is demonstrated that ethical leadership has a significant indirect relationship with followers’ emotional exhaustion through three types of emotional labor strategies; genuine display, faked display, and suppressed display. Through genuine display and suppressed display, ethical leadership had an indirect and negative relationship with followers’ emotional exhaustion, whereas ethical leadership and followers’ emotional exhaustion showed a positive indirect relationship through faked display. We discuss the implications and limitations of this research and future research directions.

## 1. Introduction

Emotional exhaustion, a chronic state of physical and emotional depletion resulting from excessive and continuous job demands and continuous hassles [1,2], is a central concern for organizations. Employees’ emotional exhaustion can come at a high cost due to outcomes such as increased turnover, absenteeism, and reduced productivity [3], as well as a detrimental effects on individual health and well-being [4]. Emotional exhaustion has also become a central focus in the public health domain. For example, in 2019, the World Health Organization classified burnout, including emotional exhaustion, as an occupational phenomenon instead of a medical condition [5,6]. In particular, during the COVID-19 pandemic, occupational stress has been found to increase for employees in various industries such as healthcare, hotels, and educational services [7,8,9,10].

In recent decades, there has been continuous growth in research on emotional exhaustion [11]; researchers have sought to identify its antecedents and consequences, e.g., [12,13]. The conservation of resources (COR) theory [14,15] has been widely adopted to investigate the antecedents of emotional exhaustion. According to COR theory, people tend to acquire, maintain, and protect valuable resources. Thus, when an employee receives social support at work, they may feel less exhausted. On the other hand, if work-related energy consumption is continual, it could result in emotional exhaustion [16]. Using COR theory, we propose that ethical leadership is associated with lowering follower emotional exhaustion due to the provision of valuable resources and prevention of resource loss.

We examine the mediating effects of followers’ emotional labor for their leaders on the relationship between ethical leadership and emotional exhaustion. Emotional labor entails the regulation of emotions according to established rules of expression (i.e., display rule) [17], and several studies have demonstrated that emotional labor is a plausible predictor of emotional exhaustion, e.g., [18,19,20,21]. Although emotional labor was initially defined in terms of the emotional control experienced by front-line employees for their customers, e.g., [22,23], research has increasingly focused on emotional labor as it emerges from coworker interactions within an organization, e.g., [24,25,26]. Furthermore, there is a strong likelihood that emotional labor by followers for their leaders frequently occurs in relationships within an organization. Leaders are generally responsible for resource allocation and performance evaluation [27], and they play an important role in followers’ work-related experiences [28]. According to Arnold et al. [29] and Humphrey [30], leaders and followers routinely regulate their emotions, and we anticipate numerous instances in which followers must manage their feelings toward leaders as a result of the leaders’ influence on them.

Following the classification of Glomb and Tews [31], we identified a link between ethical leadership and three distinct types of emotional labor (e.g., genuine display, faked display, and suppressed display). Additionally, we examined whether ethical leadership has an indirect negative relationship with the emotional exhaustion of followers via these forms of emotional labor.

In this study, we contribute to the literature on ethical leadership, emotional exhaustion, and emotional labor in three main ways. First, our research provides empirical evidence for the body of literature regarding the relationship between ethical leadership and follower well-being. Second, we identify the role of intra-organizational emotional labor as the underlying mechanism through which ethical leadership relates to followers’ outcomes. Third, we consider that our research will establish an empirical foundation for intra-organizational emotional labor, a concept whose importance is widely acknowledged but for which there is a dearth of empirical research. We consider this study to be the first to establish a link between ethical leadership, intra-organizational emotional labor, and follower emotional exhaustion. The results of this examination can help guide the prevention of employees’ emotional exhaustion.

## 2. Theoretical Background

### 2.1. Emotional Exhaustion

Emotional exhaustion is the perception that one’s energy resources are depleted [1]. It is characterized by fatigue and a sense of being worn out by work [1,2]. Emotional exhaustion is a sub-dimension of burnout, along with depersonalization and cynicism, and it is generally regarded as a core feature of burnout, e.g., [6,32,33]. Emotional exhaustion among employees has significant consequences for both individuals and organizations. Chronic work-related stress, for example, has been linked to worse cognitive functioning [34] and has significant comorbidity with anxiety and depression [35]. Emotional exhaustion also significantly predicts decreased job satisfaction [36] and lower life satisfaction [4]. In terms of human resource management, employees’ emotional exhaustion is also associated with turnover, absenteeism, lower performance, and impaired organizational citizenship behavior, with substantial repercussions for the organization [3,37]. 

A growing body of research has investigated the antecedents of emotional exhaustion in terms of resources [6,33,38]. From a resource perspective, emotional exhaustion occurs when individuals lack accessible resources. According to COR theory, individuals seek to acquire, maintain, and protect resources, and when those resources become scarce or threatened, they experience frustration, helplessness, and emotional exhaustion [39].

### 2.2. Ethical Leadership and Followers’ Emotional Exhaustion 

Ethical leadership is defined as “the demonstration of normatively appropriate conduct of through personal actions and interpersonal relationships, and the promotion of such conduct to followers through two-way communication, reinforcement, and decision-making” [40,41] (p. 120). Ethical leadership entails treating followers fairly and respectfully, along with demonstrating care for them [42]. Ethical leadership behavior also emphasizes transparency, involving employees in decision making and providing followers with a clear understanding of ethical standards and expectations. Additionally, ethical leaders explicitly reward desirable behavior and punish unethical behavior [40]. 

According to COR theory, individuals attempt to acquire, retain, protect, and foster valuable resources while avoiding threats of resource loss [14,15]. Considering ethical leadership to be a valuable resource and a mitigating factor for employee resource loss, we applied COR theory to develop the hypothesis that ethical leadership is associated with less emotional exhaustion in followers. First, an ethical leader’s care towards and fair treatment of followers, including two-way communication [40], can help alleviate stress and emotional exhaustion in employees. Prior research indicates that when individuals perceive social support, they experience less emotional exhaustion [43,44], and ethical leaders generally foster a nurturing and supportive work environment [33]. Second, ethical leaders provide clear ethical standards to their followers and model proper behavior by using a reward system (rewards and discipline). This might reduce followers’ negative feelings that can come from uncertainty when cooperating within an organization. In earlier research, ethical leadership increased followers’ perceived procedural fairness [45] and reduced perceived uncertainty [33]. Finally, when they adhere to high ethical standards, ethical leaders demonstrate consistency in their honesty and truthfulness and build trust among their followers [41,46]. Trust reduces stress in individuals and is negatively related to emotional exhaustion [47,48]. From these considerations, we hypothesized that ethical leadership is associated with a low level of emotional exhaustion in followers.

**Hypothesis** **1.** *Ethical leadership is negatively related to followers’ emotional exhaustion*.

### 2.3. Ethical Leadership and Intra-Organizational Emotional Labor

People experience emotions every day [49]. In the majority of situations, individuals generally express their emotions appropriately instead of expressing them naturally [50]. Emotional regulation is the process through which an individual controls their emotions [51]. Individuals decide how and whether to express everyday emotions and apply emotion regulation appropriately in accordance with this decision [52], which determines how they increase, sustain, or decrease positive and negative emotions [51].

Recent research has presented emotional labor as an emotion regulation process in which individuals manage their own emotions to comply with workplace norms [17,53]. Workplaces often have standards for the expression of emotion, and workers control the emotions they experience in accordance with these norms, and they regard doing so as part of their work [54]. According to Glomb and Tews [31], emotional labor can be classified into three categories: The first is termed genuine display. As genuine display also requires the use of cognitive effort to ensure that an emotion that has arisen spontaneously is appropriately presented, scholars theorize that genuine, spontaneous, and natural emotional displays that are in accordance with organizational display rules are also a form of emotional labor [22]. The second is faked display, which is an exaggerated presentation of emotions that the expresser is not experiencing. The final category is suppressed display, which describes masking emotions that occur according to display rules. 

We focused on the relationship between ethical leadership and followers’ emotional labor toward their leaders: intra-organizational emotional labor [55]. Although earlier research mostly focused on emotional labor in interactions between front-line employees and their customers, e.g., [22,23,53], emotion regulation in relation to complying with display rules is increasingly being recognized as existing in intra-organizational relations across various occupations [25,26,55]. Mann [26] and Kramer and Hess [25] found that emotional labor is often seen in interactions with coworkers. Ozcelik [55] argued that emotional labor for leaders or coworkers should be studied separately from emotional labor for customers because the display rules within an organization may not be explicit and may be subtly altered depending on the precise relationship. Glasø et al. [24] conducted an emotional labor study between leaders and followers in an organization, which presented the possibility of followers engaging in emotional labor during leader–follower interactions. Further, Arnold et al. [29] demonstrated that leadership styles such as transformational leadership affect followers’ emotional regulation strategies. 

Ozcelik [55] found that faked and suppressed displays of emotional labor increase in the following situations: (1) where there are insufficient reasonable criteria for obtaining rewards or benefits within an organization, and (2) when individuals seek to control themselves in relation to the perspective of others (i.e., self-monitoring). From Ozcelik’s [55] perspective, ethical leadership is likely to be related to followers’ emotional labor (i.e., genuine display, faked display, and suppressed display) for the following reasons: First, where ethical leaders establish clear ethical standards and adhere to them, followers are less likely to exaggerate or suppress their emotions to receive rewards or benefits from the organization. On the other hand, followers are more likely to express their emotions genuinely so long as they adhere to the leader’s declared ethical standard. Second, ethical leaders show care for their followers [40], which reduces followers’ propensity for self-monitoring and, as a result, lowers followers’ faked and suppressed displays. Thus, it is plausible to expect that consideration by an ethical leader would lead to a genuine display by their followers. In conclusion, followers are expected to engage in more genuine and less faked and suppressed displays when interacting with ethical leaders. Accordingly, we formed the following hypotheses.

**Hypothesis** **2a.** *Ethical leadership is positively related to followers’ genuine display*.

**Hypothesis** **2b.** *Ethical leadership is negatively related to followers’ faked display*.

**Hypothesis** **2c.** *Ethical leadership is negatively related to followers’ suppressed display*.

### 2.4. The Mediating Effect of Emotional Labor

Finally, we anticipated the mediating roles of the three types of emotional labor in the relationship between ethical leadership and the emotional exhaustion of followers. From the COR perspective, employees are confronted with the emotional demands of their roles (e.g., display rules). In response to such demands, they devote resources (i.e., emotional labor) in anticipation of developing rewarding relationships with their customers, clients, leaders, or coworkers [16]. According to Brotheridge and Lee [16], when individuals adopt emotionally taxing emotional labor tactics, they may experience extreme exhaustion. On the other hand, it is acknowledged that individuals experience less exhaustion when they adopt less demanding emotional labor tactics, or when they are more likely to acquire a positive experience, such as accomplishment and specific self-efficacy following emotional labor [16,38]. In earlier research, e.g., [50,53], faked and suppressed displays were reported to be response-focused types of emotion regulation, which require a substantial level of emotional resources. As a result, it has been demonstrated that both faked and suppressed displays can have positive effects on emotional exhaustion, e.g., [56,57]. However, although genuine display is an emotional labor strategy, it consumes fewer resources because a genuine display strategy does not attempt to correct already encountered emotions [50,53]. Furthermore, as the strategy of genuine display unifies internal and expressive emotions, it is more likely to produce a sense of accomplishment or a positive self-evaluation than faked or suppressed display would [17,38]. Thus, genuine display generally decreased emotional exhaustion in previous studies [19,58,59].

Taken together, we assumed that ethical leadership is likely to be positively associated with followers’ genuine displays (Hypothesis 2), and these genuine displays may help alleviate their emotional exhaustion. In other words, ethical leadership is likely to show an indirect negative relationship with followers’ emotional exhaustion through the genuine displays of followers. Meanwhile, ethical leadership has a negative relationship with followers’ faked and suppressed displays (Hypotheses 2b and 2c), which seems to aggravate their emotional exhaustion; ethical leadership has a negative indirect relationship with emotional exhaustion via the faked and suppressed displays of followers. Thus, we formed the following hypotheses.

**Hypothesis** **3a.** *Followers’ genuine display mediates the relationship between ethical leadership and followers’ emotional exhaustion*.

**Hypothesis** **3b.** *Followers’ faked display mediates the relationship between ethical leadership and followers’ emotional exhaustion*.

**Hypothesis** **3c.** *Followers’ suppressed display mediates the relationship between ethical leadership and followers’ emotional exhaustion*.

## 3. Materials and Method

### 3.1. Participants and Procedure

The participants were employees from various organizations in South Korea. They were recruited through a research company that administered an online survey. The study surveyed 343 individuals, of whom 295 responded to all questions. Omitting careless responses, a total of 259 (87.8%) responses were used in the analysis. 

Of the 259 participants, 46.7% were female. The average age of the participants was 36.49 years (SD = 7.93), with a range from 20 to 60 years. All participants had been working for more than 6 months at their current workplace and had a direct work relationship with a leader. Their average organizational tenure was 11.45 years (SD = 0.88). A total of 72% had graduate-level education, 15% were community college graduates, and 12% were high school graduates.

### 3.2. Measures

#### 3.2.1. Ethical Leadership

Ethical leadership was measured with a 10-item scale developed by Brown et al. [41]. Sample items include “My leader sets an example of how to do things the right way in terms of ethics” and “My leader conducts his or her personal life in an ethical manner.” Items were rated on a 5-point Likert scale ranging from 1 (totally disagree) to 5 (totally agree). The Cronbach α value for the scale in [41] was 0.90, and the value obtained for this study was 0.91. 

#### 3.2.2. Emotional Labor 

Emotion regulation strategies were measured using the DEELS (Discrete Emotions Labor Scale) developed by Glomb and Tews [31]. The DEELS includes three subscales representing genuine display, faked display, and suppressed display. Each subscale includes four discrete emotions, as proposed by Glasø and Einarsen [60]. 

For each subscale, respondents are asked to consider each emotion in relation to their interactions with leaders over the period of 1 month. For the genuine display subscale, respondents were asked, “How often do you genuinely express (inspiration/enthusiasm/ interest/excitement) when you feel it?” In the faked display subscale, respondents were asked, “How often do you express feelings of (inspiration/enthusiasm/ interest/excitement) on the job when you really don’t feel that way?” For the suppressed display subscale, respondents were asked, “How often do you keep (annoyance/frustration/worry/ boredom) to yourself when you really feel that way?” Items were rated on a 5-point Likert scale ranging from 1 (never) to 5 (many times a day). The Cronbach α values for these subscales were 0.83 for genuine display, 0.86 for faked display, and 0.88 for suppressed display. 

#### 3.2.3. Emotional Exhaustion 

This was measured using the emotional exhaustion scale from the Maslach Burnout Inventory [61]. This scale included 5 items (e.g., “I feel emotionally drained from my work”) and used a 5-point Likert scale (1 = totally disagree, 5 = totally agree). Maslach et al. [61] reported a Cronbach α of 0.92 for their scale, and the Cronbach α was 0.86 in the current study. 

### 3.3. Analytical Strategy 

First, we used IBM SPSS 25 to estimate the means, standard deviations, internal consistency coefficients, and correlations between variables. Next, for hypothesis testing, we conducted regression with SPSS 25, and structured equation modeling (SEM) was performed with MPlus 7.4 [62]. Regression analysis was conducted to test total effects [63] of ethical leadership on followers’ emotional exhaustion. Then, SEM was applied to estimate the structural paths to test the hypothesized relationship between the constructs. All paths, including indirect effects, were estimated using bootstrapping with 10,000 iterations and a 95% confidence interval. 

## 4. Results

### 4.1. Descriptive Statistics

Means, standard deviations, and two-tailed zero-order correlations of the variables are reported in Table 1. 

### 4.2. Measurement Model Analysis

Because all of our measures were addressed by the same source, we conducted a series of confirmatory factor analyses to test the distinctiveness of the constructs. We compared the measurement model (Model 0: theorized five-factor model) with two nested models. The first was a three-factor model, in which three mediation variables (genuine display, faked display, and suppressed display) were merged into one factor (Model 1). The second model was a one-factor model (Model 2). 

As shown in Table 2, the results demonstrate that the theorized five-factor model (ethical leadership, genuine display, faked display, suppressed display, and emotional exhaustion) provided a fair fit to the data, χ^2^ = 537.11; df = 242; CFI = 0.92; TLI = 0.91; RMSEA = 0.06. Additionally, the results of the fit index and the chi-square difference test indicate that the five-factor model fits the data better than the alternative models. 

### 4.3. Hypothesis Tests

Hypothesis 1 proposed that ethical leadership is negatively associated with followers’ emotional exhaustion. According to linear regression, ethical leadership had a significant and negative relationship with followers’ emotional exhaustion, after controlling for gender, age, education, and tenure (*b* = −0.16, *SE* = 0.07, *p* < 0.05). Thus, Hypotheses 1 was supported. 

**Figure 1:**
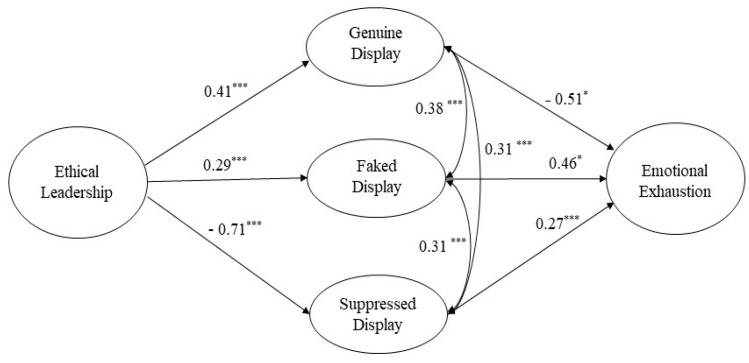
Structural model with standardized effects. Note. * *p* < 0.05, *** *p* < 0.001, fit indices: χ^2^ = 664.84 (df = 335); CFI = 0.91; TLI = 0.90; RMSEA = 0.06.

Then, we estimated the direct and indirect paths of the study using SEM. Based on Glomb and Tews [31] demonstrating that the three types of emotional labor are associated, we established inter-correlations between the three types and examined our hypothesized structure model. As shown in Figure 1 and Table 3, the relationship between ethical leadership and genuine display (Hypothesis 2a) was positive and significant (*b* = 0.41, SE = 0.11, *p* < 0.001). Additionally, the relationship between ethical leadership and suppressed display (Hypothesis 2c) was negative and significant (*b* = −0.71, SE = 0.13, *p* < 0.001). Thus, Hypotheses 2a and 2c were supported. Meanwhile, contrary to our hypothesis, the relationship between ethical leadership and faked display was significant and positive (*b* = 0.29, SE = 0.11, *p* < 0.01). Thus, Hypothesis 2b was not supported.

Hypothesis 3a predicted that ethical leadership would have a negative indirect effect on emotional exhaustion through followers’ genuine display of emotional labor. As shown in Table 3, the indirect association between ethical leadership and followers’ emotional exhaustion through followers’ genuine display was negative and significant (indirect effect = −0.21, 95% CI [−0.56, −0.04]). High levels of ethical leadership have a significant relationship with followers’ low emotional exhaustion because followers experience a high level of genuine display. Thus, Hypothesis 3a was supported. 

Next, as suggested in Hypothesis 3c, the indirect relationship between ethical leadership and followers’ emotional exhaustion through suppressed display was negative and significant (indirect effect = −0.19, 95% CI [−0.31, −0.10]). Followers who considered their leader to be ethical have less control over their emotions and reported low emotional exhaustion. Thus, Hypothesis 3c was supported. However, the result also shows that the indirect relationship between ethical leadership and followers’ emotional exhaustion via faked display was significantly positive (indirect effect = 0.13, 95% CI [0.02, 0.43]). We predicted that followers’ faked display would have a negative mediating effect between ethical leadership and followers’ emotional exhaustion. However, the results show that as the leader was more ethical, followers were more likely to use faked display. In turn, the followers experienced more emotional exhaustion. Thus, Hypothesis 3b was not supported. 

## 5. Discussion

This study aimed to examine the relationship between ethical leadership, followers’ emotional labor toward leaders, and emotional exhaustion, based on the COR theory. The COR theoretical framework, which systemically explains emotional exhaustion [2], postulates that people are motivated to conserve resources and avoid losing them. From this perspective, we sought to investigate how ethical leadership can help followers retain their resources and avoid resource loss. 

As in previous research, e.g., [6,42], our results suggest that ethical leadership can serve as a situational factor for reducing followers’ emotional exhaustion. Moreover, we identified a link between ethical leadership and followers’ emotional labor in daily organizational life, mediating the relationship between ethical leadership and followers’ emotional exhaustion. In this study, we found that followers who perceive their leaders to be ethical reported lower emotional exhaustion as a result of more genuine display and less inhibition of negative emotions.

In the case of faked display, we initially predicted that ethical leadership would re-duce faked display which is known to be positively related to followers’ emotional exhaustion, resulting in a negative and indirect effect on followers’ emotional exhaustion. However, our findings indicate that when ethical leadership is associated with followers’ faked display, this emotional labor tactic could be leveraged to increase emotional exhaustion. Given that a faked display is a fabrication of positive emotions that the individual does not actually feel [31], we interpreted this study’s findings as follows: According to previous studies dealing with the ethical leadership and organizational behaviors of followers based on social exchange theory [64], followers are more likely to show positive expressions and attitudes when they believe their leaders are ethical [65,66]. As a result, it is possible that followers try to show fabricated feelings such as passion, enthusiasm, and pleasure in order to express that they have a favorable attitude toward the leader, even though they do not experience these emotions.

Another possibility is the too-much-of-a-good-thing (TMGT) effect. Numerous recent studies have identified the TMGT effect, in which an excess of ethical leadership can result in undesirable outcomes, such as increased conformity and less creativity among followers, e.g., [67,68]. In the TMGT effect, when followers consider their leaders to exhibit a very high level of ethical leadership, they consider the standards set by their leaders as unattainable. As a result, as demonstrated by the findings of this study, it is possible that emotions are exaggerated beyond reality in order to show an attitude consistent with a leader’s expectations. Taking these explanations together, we raise the possibility that under ethical leadership, followers may experience emotional exhaustion, an unexpected result, by expressing positive emotions more than that they actually experience them to demonstrate a favorable attitude toward the leader.

### 5.1. Theoretical Implications

Our study contributes the following theoretical insights. First, using resource-based stress theory (specifically, COR theory), we explained the roles of ethical leadership and intra-organizational emotional labor as antecedents of employee emotional exhaustion. Our findings demonstrate that ethical leadership can be a compelling predictor of emotional exhaustion from a resource standpoint. Furthermore, by examining how emotional labor, which has been shown to affect emotional exhaustion in previous research, is associated with ethical leadership, we empirically examined various possible ways through which ethical leadership could affect emotional exhaustion in followers.

Second, this study identified a specific mechanism for the relationship between ethical leadership and followers’ low level of occupational stress in the workplace. Brown and Trevino [40] proposed developing and empirically validating a new underlying mechanism between ethical leadership and follower outcomes, in addition to mechanisms based on social learning theory [69] and social exchange theory [64] that have been presented in previous research (e.g., identification, commitment). Using COR theory, we propose emotional labor as a new mechanism that links ethical leadership and followers’ emotional exhaustion. Our study contributes a novel perspective to the field of ethical leadership research and enriches previous empirical findings. 

Finally, our findings contribute to the growing body of literature on intra-organizational emotional labor, which remains devoid of empirical evidence. In this study, we demonstrated the discriminant validity of three emotional labor strategies (genuine display, faked display, and suppressed display) in the context of intra-organization. Additionally, in support of Arnold et al. [29], we confirmed that leadership styles show a significant relationship with emotional labor directed at leaders. This study contributes to the field of emotional labor in organizations, which, in spite of its constant growth, has continued to provide insufficient empirical evidence to date. 

### 5.2. Practical Implications

This study also has implications for practice. First, for organizations and HR personnel, our findings suggest that they should encourage leaders to understand and practice ethical leadership behavior. This study found that ethical leadership has a considerable positive effect on employee well-being such as positive affective states, happiness, and meaningfulness at work [70,71]. Thus, the behavioral features observed in ethical leaders may be able to mitigate followers’ emotional depletion. 

Second, our findings suggest that leaders should acknowledge that their followers perform various types of emotional labor while interacting with them. Furthermore, as emotional labor between leaders and followers occurs regularly in organizations, leaders should recognize the reality of their followers’ emotional labor and foster an organizational environment in which followers can express themselves genuinely instead of suppressing or faking their emotions.

### 5.3. Limitations and Future Research 

This study has several limitations, and it opens possible directions for future research. First, this study was conducted among office workers in South Korea, a highly specific population; it is necessary to take caution when generalizing the results of this study. In the case of emotional labor, which was explored as a mediator in this study, earlier research, e.g., [72,73], showed that employees’ emotional labor strategies might differ between Eastern cultures consisting of China, Japan, and South Korea and Western cultures such as the US and Canada. Thus, future research will be able to validate our study repeatedly in various countries and cultures to ascertain how the antecedents and mechanisms discovered in our study appear in other cultures. 

Second, although we identified the discriminant validity of the hypothesized factors and recognized that the risk of common method bias was low, we collected all variables via self-reporting from a single source. Future study could be conducted with separate investigations for separate groups of respondents, with leaders assessing leadership factors and followers assessing emotional labor and exhaustion. 

Finally, this study employed a cross-sectional design. As a result, we could not establish a causal relationship between variables. Future research should apply longitudinal or experimental designs to better describe the relationships among ethical leadership, emotional labor, and emotional exhaustion that were identified in this study.

## 6. Conclusions

Using COR theory, this study examined the relationships among ethical leadership, followers’ intra-organizational emotional labor, and emotional exhaustion. This study identified that ethical leadership was negatively related to followers’ emotional exhaustion and was indirectly associated with followers’ exhaustion through genuine and suppressed displays of emotional labor strategies. As a result of our findings, we propose that ethical leadership can effectively seek to alleviate followers’ emotional exhaustion. This study made the theoretical contribution of introducing a mechanism for followers’ emotional labor for leaders to the relationship between ethical leadership and the emotional exhaustion of followers. Further, this research provides practical implications for organizations and their leaders. Future research can further generalize the results obtained here and investigate the mechanism that this study discovered through the use of a longitudinal study or an experimental design involving people from diverse cultural backgrounds.

## Figures and Tables

**Table 1:** Descriptive statistics, and correlations among variables (*N* = 259).

	*M*	*SD*	1	2	3	4	5	6	7	8
1. Gender	0.47	0.50								
2. Age	36.49	7.93	−0.16 *							
3. Education	2.69	0.79	−0.06	−0.14 *						
4. Tenure	11.45	0.88	0.02	0.38 **	0.03					
5. Ethical leadership	3.23	0.70	−0.07	0.07	0.08	−0.03				
6. Genuine display	2.50	0.84	−0.12	0.00	0.03	0.01	0.34 **			
7. Faked display	2.45	0.85	−0.10	0.00	0.00	0.03	0.24 **	0.76 **		
8. Suppressed display	2.18	0.99	−0.06	0.03	−0.08	0.07	−0.30 **	0.27 **	0.32 **	
9. Emotional exhaustion	3.26	0.81	0.14 *	−0.08	−0.06	−0.01	−0.15 *	0.04	0.14 *	0.30 **

*Note.* * *p* < 0.05, ** *p* < 0.01 (two-tailed), gender: 0 = man, 1 = woman.

**Table 2:** Comparison of measurement models.

Models	Factor	χ^2^	df	Δχ^2^	CFI	TLI	RMSEA
0	Theorized five-factor model	537.11	242	-	0.92	0.91	0.06
1	Three-factor model	1045.94	249	508.83 ***	0.78	0.76	0.11
2	One-factor model	2391.36	252	1345.42 ***	0.41	0.35	0.18

*Note*. *** *p* < 0.001, Theorized five-factor model: ethical leadership, genuine display, faked display, suppressed display, emotional exhaustion; Three-factor model: ethical leadership, emotion regulation, emotional exhaustion.

**Table 3:** Summary of standardized direct and indirect effects.

**Standardized Direct Effect**	**Estimates**	**SE**	**95% CI**
Ethical leadership → Genuine display	0.41	0.11	[0.25, 0.62]
Ethical leadership → Faked display	0.29	0.11	[0.13, 0.48]
Ethical leadership → Suppressed display	−0.71	0.13	[−0.93, −0.50]
Genuine display → Emotional exhaustion	−0.51	0.53	[−1.20, −0.07]
Faked display → Emotional exhaustion	0.46	0.50	[0.03, 1.12]
Suppressed display → Emotional exhaustion	0.27	0.07	[0.16, 0.38]
**Standardized Indirect Effect**	**Estimates**	**SE**	**95% CI**
EL → GD → EE	−0.21	0.22	[−0.56, −0.04]
EL → FD → EE	0.13	0.18	[0.02, 0.43]
EL → SD → EE	−0.19	0.06	[−0.31, −0.10]

*Note.* *N* = 259, SE = standard error, CI = confidence interval, EL = ethical leadership, GD = genuine display, FD = faked display, SD = suppressed display, EE = emotional exhaustion.

## Data Availability

The data presented in this study are available on request from the corresponding author. The data are not publicly available due to privacy issues.
